# Current Oncology: A Multidisciplinary Medium for Clinical Oncology

**DOI:** 10.3390/curroncol28010002

**Published:** 2020-11-28

**Authors:** Sharlene Gill

**Affiliations:** Department of Medicine, Faculty of Medicine, University of British Columbia, Vancouver, BC V5Z 4E6, Canada; sgill@bccancer.bc.ca

In recent years, the field of oncology has witnessed the unprecedented pace of genomics discovery, knowledge translation, and clinical research validation, which has led to novel systemic and locoregional therapeutics, an evolving role of immunotherapy, and a broader application of precision oncology. At the same time, we are gaining a greater appreciation for adaptive trial designs, real-world evidence, and patient-reported outcomes to better inform the optimal utilization of new therapies which offer the greatest benefit to our patients for the greatest value to society.

Within this era of exciting developments, I am delighted to assume the role of Editor-in-Chief for *Current Oncology* [1]—a scientific, peer-reviewed open access journal established in 1994 in Canada, and now published bi-monthly by MDPI (Basel, Switzerland). This journal provides a platform to disseminate high-quality, original clinical research, expert reviews, and periodic Special Issues—with the clinical oncologist and healthcare provider audience in mind. *Current Oncology* is committed to being recognized as an outstanding clinical oncology journal with rigorous peer review and an efficient publication process.

On behalf of the Editorial Board and team, I welcome you to *Current Oncology* and encourage you to submit your original research works to the journal. We look forward to your contributions and continued engagement.
